# New balance capability index as a screening tool for mild cognitive impairment

**DOI:** 10.1186/s12877-023-03777-6

**Published:** 2023-02-04

**Authors:** Yasuhiro Suzuki, Takumi Tsubaki, Kensuke Nakaya, Genta Kondo, Yoshinori Takeuchi, Yuichi Aita, Yuki Murayama, Akito Shikama, Yukari Masuda, Hiroaki Suzuki, Yasushi Kawakami, Hitoshi Shimano, Tetsuaki Arai, Yasushi Hada, Naoya Yahagi

**Affiliations:** 1 JST START University Ecosystem Promotion Type (University Promotion Type) Project Team, Headquarters for International Industry-University Collaboration, University of Tsukuba, 1-1-1 Tennodai, Tsukuba, Ibaraki 305-8575 Japan; 2 Department of Rehabilitation Medicine, University of Tsukuba Hospital, Ibaraki, 305-8596 Japan; 3 Nutrigenomics Research Group, Institute of Medicine, University of Tsukuba, Ibaraki, 305-8575 Japan; 4 Department of Internal Medicine (Endocrinology and Metabolism), Institute of Medicine, University of Tsukuba, Ibaraki, 305-8575 Japan; 5 Department of Laboratory Medicine, Institute of Medicine, University of Tsukuba, Ibaraki, 305-8575 Japan; 6 Department of Psychiatry, Institute of Medicine, University of Tsukuba, Ibaraki, 305-8575 Japan

**Keywords:** Mild cognitive impairment (MCI), Alzheimer's disease (AD), Vestibular function, Balance, Stabilometer, Postural stability

## Abstract

**Background:**

Mild cognitive impairment (MCI) is not just a prodrome to dementia, but a very important intervention point to prevent dementia caused by Alzheimer's disease (AD). It has long been known that people with AD have a higher frequency of falls with some gait instability. Recent evidence suggests that vestibular impairment is disproportionately prevalent among individuals with MCI and dementia due to AD. Therefore, we hypothesized that the measurement of balance capability is helpful to identify individuals with MCI.

**Methods:**

First, we developed a useful method to evaluate balance capability as well as vestibular function using Nintendo Wii balance board as a stabilometer and foam rubber on it. Then, 49 healthy volunteers aged from 56 to 75 with no clinically apparent cognitive impairment were recruited and the association between their balance capability and cognitive function was examined. Cognitive functions were assessed by MoCA, MMSE, CDR, and TMT-A and -B tests.

**Results:**

The new balance capability indicator, termed visual dependency index of postural stability (VPS), was highly associated with cognitive impairment assessed by MoCA, and the area under the receiver operating characteristic (ROC) curve was more than 0.8, demonstrating high sensitivity and specificity (app. 80% and 60%, respectively).

**Conclusions:**

Early evidence suggests that VPS measured using Nintendo Wii balance board as a stabilometer helps identify individuals with MCI at an early and preclinical stage with high sensitivity, establishing a useful method to screen MCI.

**Supplementary Information:**

The online version contains supplementary material available at 10.1186/s12877-023-03777-6.

## Background

Approximately 50 million people live with dementia worldwide, and this number is predicted to increase to 152 million by 2050 [1]. Total cost in 2019 for people with dementia was estimated to be $1.3 trillion globally, which could rise to $1.7 trillion by 2030 [2]. Thus, the burden of dementia continues to grow, and prevention strategies including early identification and early treatment of the disease are highly needed.

Although our understanding of dementia etiology is still shifting, Alzheimer's disease (AD) is the most common type of dementia, accounting for an estimated 60% to 80% of cases [3]. The progression of AD is called the AD continuum. On this continuum, there are three broad phases: preclinical AD, mild cognitive impairment (MCI) due to AD and dementia due to AD [3]. A meta-analysis study found that among individuals with MCI who were tracked for 5 years or longer, 38% developed dementia [4]. However, in some individuals MCI reverts to normal cognition or remains stable, particularly with some appropriate intervention [1]. Therefore, it is important to identify individuals with MCI early in the course of AD in order to protect from dementia.

Generally, individuals with dementia are vulnerable for a decline in physical functioning and basic activities of daily living [5]. In fact, as a characteristic of cognitive impairment and dementia, it has long been known that people with AD have a higher frequency of falls [6, 7], and today, cognitive impairment is well established as an independent risk factor for falling [8, 9]. Moreover, emerging evidence indicates that early disturbances in cognitive function are associated with slower gait and gait instability [10, 11]. Given that gait and balance are deeply connected [12], some relationship between cognitive function and balance capability can be implicated.

The vestibular (inner ear balance) system senses head movement and orientation in space, and vestibular sensory input plays a critical role in postural control, contributing to balance capability which can be measured by static postural balance parameters during standing [13]. Intriguingly, growing evidence suggests that vestibular impairment is disproportionately prevalent among individuals with MCI and dementia due to AD [14, 15].

Posture in human is maintained using sensory inputs critical for balance, namely vestibular, visual, and somatosensory inputs [16]. Posturography is a technique used to quantify postural control in upright stance on a stabilometer. Recently, we and others have developed a useful method to evaluate balance capability as well as vestibular function using a stabilometer and foam rubber [13, 17–19]. With further modifications to this method, we hypothesized that we can distinguish between people with MCI and healthy people using a stabilometer. Here we report that a new balance capability indicator named visual dependency index of postural stability (VPS) is highly associated with cognitive impairment and helps screen individuals with MCI. Thus, VPS measurement using Wii balance board (WBB) can be an inexpensive MCI screening system.

## Methods

### Participants

From December 2020 to February 2021, we enrolled 49 participants with healthy conditions who were 56 to 75 years old and had no clinically apparent cognitive impairment. The participants were recruited using our department’s website, bulletin boards at our university, and a local community magazine. Participants were paid a reward of 7,000 yen. Exclusion criteria included (i) history of chronic diseases (dementia, diabetes, kidney disease, collagen disease, peripheral neuropathy); (ii) having a disability certificate or using long-term care insurance; (iii) being unable to walk independently without assistive devices (including transportation). No one was excluded based on results of cognitive function tests. Written informed consent was obtained from participants prior to study enrollment. This study was conducted in accordance with the principles of the Declaration of Helsinki as well as the Ethical Guidelines for Medical and Health Research Involving Human Subjects in Japan and approved by the Clinical Research Ethics Committee of University of Tsukuba Hospital (R02-251).

### Clinical evaluation

Participants were surveyed for age, sex, exercise habitation, alcohol drinking habitation, working, driving. Body composition was evaluated by bioelectrical impedance analysis (InBody 720, BioSpace, Tokyo). Body mass index (BMI) and skeletal muscle mass index were calculated by dividing the body weight (kg) by the square of the height (m^2^) and dividing the limb skeletal muscle mass (kg) by the square of the height (m^2^), respectively.

### Assessment of cognitive function

Global cognitive function was assessed using the Japanese version of the Montreal Cognitive Assessment (MoCA) [20, 21] and the Mini-Mental State Examination (MMSE) [22]. Based on MoCA, participants were categorized into two groups of normal (MoCA ≥ 26) and MCI (MoCA ≤ 25) [20]. Trail Making Test parts A and B (TMT-A/-B) were used for assessing processing speed and executive function, respectively [23]. Clinical dementia rating (CDR) were used as an observational scale to assess the severity of dementia [24–26]. These tests were selected because they are the most commonly used tests to diagnose MCI [27].

### Assessment of balance capabilities

Balance capabilities were assessed by the index of postural stability (IPS) and the visual dependency index of postural stability (VPS). IPS and VPS were measured using a stabilometer as described previously [18]. As a stabilometer, GP-6000 gravicorder (Anima Co., Tokyo, Japan) and Wii balance board (WBB; Nintendo Co, Kyoto, Japan) were used. Briefly, IPS was measured as follows; first, the participants stood in a resting position with the inside of the foot at a distance of 10 cm on the stabilometer to measure the instantaneous fluctuations in the center of pressure (COP). Then, participants were instructed to incline the body to the front, rear, right and left keeping the body straight and without moving the feet for 10 s at each position, and the instantaneous fluctuations in COP were recorded. IPS was calculated as log [(area of stability limit + area of postural sway) / area of postural sway]. Area of stability limit was calculated as the front and rear center movement distance between anterior and posterior positions × the distance between right and left positions. Area of postural sway was calculated as average measurement value in 10 s under anterior, posterior, right, left and center positions. The area of postural sway was calculated as the mean sway area of the 5 positions [28]. Visual dependency index of postural stability (VPS) was defined as the ratio of eye-opened *vs.* eye-closed IPS values measured on foam rubber. VPS values are age-adjusted by the following formula: age-adjusted VPS = V / V’, where

V (measured VPS) = eye-opened IPS / eye-closed IPS, V’ (age-predicted VPS) = O / C, O (age-predicted eye-opened IPS) = -0.0003 × age^2^ + 0.0145 × age + 1.1602 (S1 Fig) and C (age-predicted eye-closed IPS) = -0.00006 × age^2^—0.0037 × age + 0.8805 (S2 Fig). That is, the age-adjusted VPS is calculated as the actual VPS divided by age-predicted VPS, and the age-predicted VPS is estimated based on the previous data collected from healthy individuals (*n* = 256) shown in S1 and S2 Figs.

For a measurement using WBB, WBB was connected wirelessly with a Bluetooth adapter to a laptop computer. Raw data were collected simultaneously, stored and processed using custom-written software (Penguin system Co, Ibaraki, Japan).

### Statistical analyses

Based on distribution, continuous variables were expressed as deviation or median (interquartile range) and compared using the unpaired t-test or the Mann–Whitney test for two-group comparisons. We used t-test in case the normality distribution of the data was confirmed by the Shapiro–Wilk test and otherwise used the Mann–Whitney test. Categorical variables are expressed as numerals and percentages and were compared with Fisher’s exact test. Spearman’s rank correlation coefficient was used to examine bivariate associations between tests of cognitive function and balance capability. Pearson's correlation coefficient was used to examine bivariate associations between IPS of the GP-6000 gravicorder and that of WBB. Regarding the receiver operating characteristic (ROC) analysis, the VPS value and 01 classification (0 for Normal group, 1 for MCI group) for the presence or absence of MCI, the cutoff value, sensitivity, specificity, and area under of the curve (AUC) for the presence or absence of MCI were calculated. The cutoff value was decided by the point where the Youdem Index reaches the maximum value. Statistical analyses were performed using SPSS Statistics 26 (Chicago, IL, USA). Statistical significance was considered at a value of < 0.05.

## Results

### Wii balance board (WBB) has the same performance as an authentic stabilometer

**Fig. 1:**
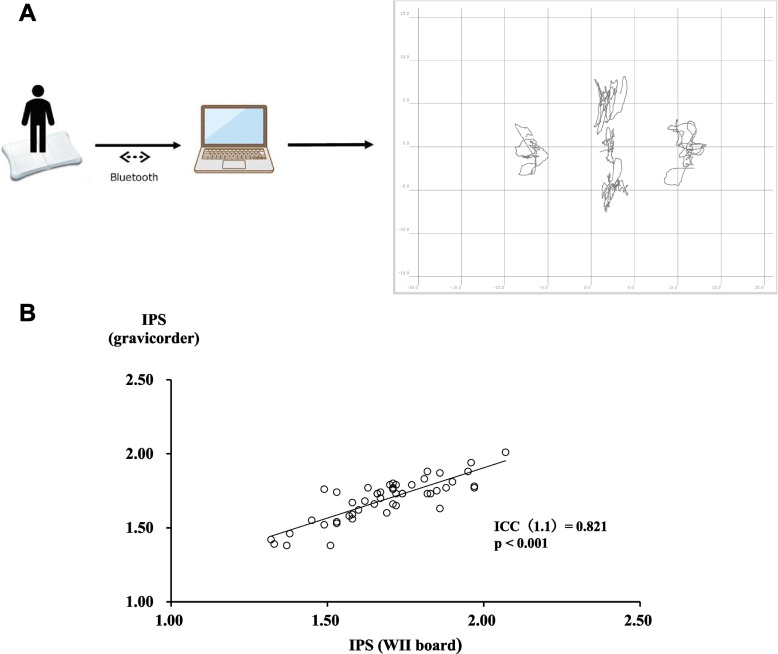
WBB has the same performance as an authentic stabilometer. **A** Schematic representation of the IPS measurement system using WBB. **B** Correlation between IPS values measured by an authentic stabilometer (GP-6000 gravicorder) and those by WBB is shown (*n* = 49). IPS, index of postural stability; VPS, visual dependency index of postural stability; WBB, Wii balance board

Because the final goal of our project is to implement an inexpensive MCI screening system to provide the best opportunity for interventions to prevent dementia, we started from developing a new posturographic system utilizing Wii balance board (WBB), a Nintendo game machine available at a very low cost (lower than $100). According to several previous reports where WBB was tested in a clinical setting to track and record the center of pressure (COP) of subjects [29–31], we developed a new software run on windows PC to measure and calculate index of postural stability (IPS) values (Fig. 1A) [17, 18]. As expected, WBB showed a good performance to measure IPS values as accurately as an authentic stabilometer for medical use (GP-6000 gravicorder), as shown in Fig. 1B.

### VPS and cognitive function show a significant relationship

Next, we devised a new indicator of postural stability that is more associated with vestibular function, based on the previous reports: combination of IPS [17] and utilization of foam rubber as well as eye closure [13, 18, 32]. The resultant new indicator, named visual dependency index of postural stability (VPS), was defined as the ratio of eye-opened *vs.* eye-closed IPS values measured on foam rubber.

**Fig. 2:**
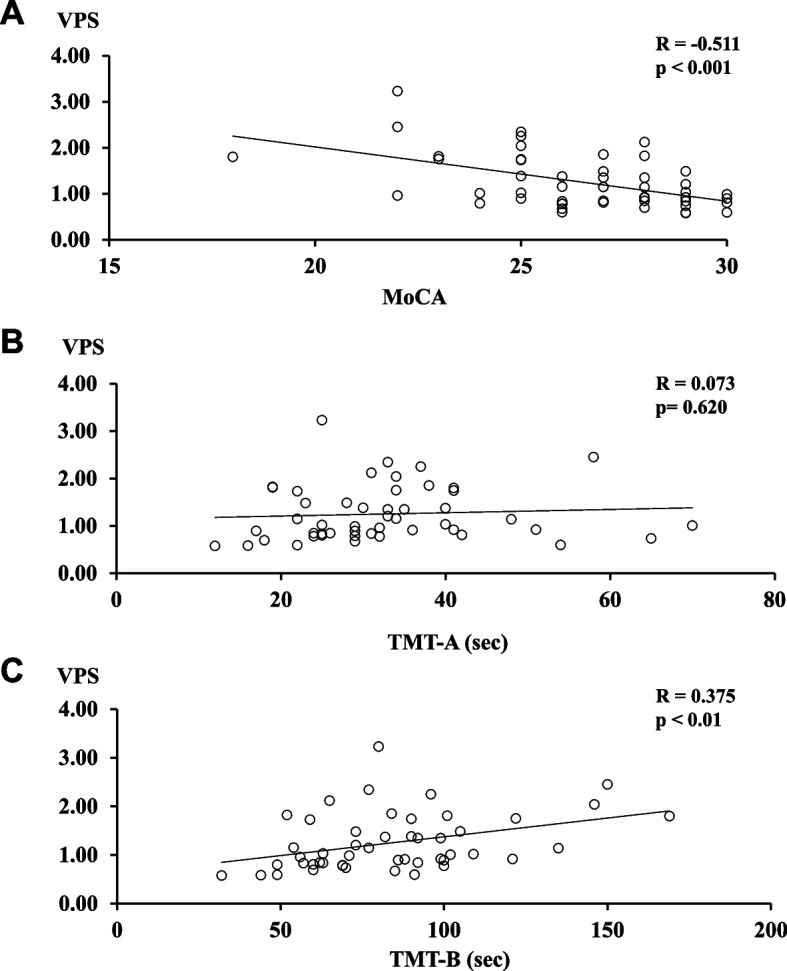
VPS and cognitive function show a significant relationship. Correlation between test VPS and cognitive function is shown. Cognitive function is assessed by **A** MoCA, **B** TMT-A and **C** TMT-B. VPS, visual dependency index of postural stability; MoCA, Montreal cognitive assessment; TMT-A, trail making test part A; TMT-B, trail making test part B

Using VPS, we examined the possible association between vestibular function and cognitive impairment. As a stabilometer, WBB was used with our original software. Table 1 showed the clinical characteristics of the study participants, basically healthy volunteers (*n* = 49). Japanese version of the Montreal Cognitive Assessment (MoCA) was used to assess cognitive function [20, 21]. It is widely accepted that MoCA is superior to MMSE in the detection of MCI as the MMSE has lower sensitivity [21, 33]. In fact, as shown in Table 2, both the normal and MCI groups diagnosed based on MoCA showed nearly full score (30 points) on MMSE. As shown in Fig. 2A, VPS values exhibited a significant negative correlation with MoCA scores (*R* = -0.511; *p* < 0.001). Also, significant positive association was observed between VPS and TMT-B scores (*R* = 0.375; *p* < 0.01), another scoring method to evaluate cognitive function (Fig. 2B, C).

**Table 1:** Clinical characteristics of participants

	All (*n* = 49)	Normal (*n* = 33) [95%CI]	MCI (*n* = 16) [95%CI]	*P*
Age (years)	66 ± 6	65 ± 6	69 ± 5	NS^#^
Female sex, n (%)	23 (47)	18 (55)	5 (31)	NS^#^
Body mass index (kg/m^2^)	22.3 ± 2.9	22.0 ± 2.8 [20.3–23.8]	22.8 ± 2.8 [21.3–24.4]	NS*
Body fat percentage (%)	26.4 ± 6.8	26.5 ± 6.7 [22.4–30.4]	26.3 ± 7.2 [22.2–30.5]	NS*
Skeletal muscle percentage (%)	40.0 ± 4.2	39.9 ± 4.0 [37.9–42.7]	40.3 ± 4.6 [37.7–42.9]	NS*
Current Exercise habitation, n (%)	25 (51)	17 (52)	8 (50)	NS^#^
Current Alcohol Drinking, n (%)	25 (51)	16 (48)	9 (56)	NS^#^
Current Working, n (%)	34 (69)	21 (64)	13 (81)	NS^#^
Current Driving, n (%)	48 (99)	32 (97)	16 (100)	NS^#^

Data are mean ± SD

*NS* Not significant, *MCI* Mild cognitive impairment

Test statistic used: *t-test, ^#^Mann–Whitney test

**Table 2:** Comparison of cognitive function tests

	All (*n* = 49)	Normal (*n* = 33) [95%CI]	MCI (*n* = 16) [95%CI]	Mean difference [95%CI]	*P*
MoCA (points)	27 (25 – 28)	28 (27 – 29)	25 (23 – 25)	—	< 0.0001^#$^
MMSE (points)	30 (29 – 30)	30 (29 – 30)	29 (29 – 30)	—	NS^#^
CDR (points)	0 (0 – 0)	0 (0 – 0)	0 (0 – 0)	—	NS^#^
TMT-A (sec)	31.0 (25.0 – 38.0)	31.0 (24.0–38.0)	32.5 (28.0–38.0)	—	NS^#^
TMT-B (sec)	81.0 (62.8 – 99.0)	73.0 (60.0–91.0) [57.4–78.6]	96.0 (78.5–105.5) [80.8–114.7]	-25.2 [-43.8 – -6.6]	0.010*^$^

Data are median (interquartile range)

*MCI* Mild cognitive impairment, *MoCA* Montreal cognitive assessment, *MMSE* Mini-mental state examination, *CDR* Clinical dementia rating, *TMT-A* Trail making test part A, *TMT-B* Trail making test part B

Test statistic used: *t-test, ^#^Mann–Whitney test. ^$^Significant at *p* < 0.05. *NS* Not significant

### MCI group has significantly higher VPS

**Fig. 3:**
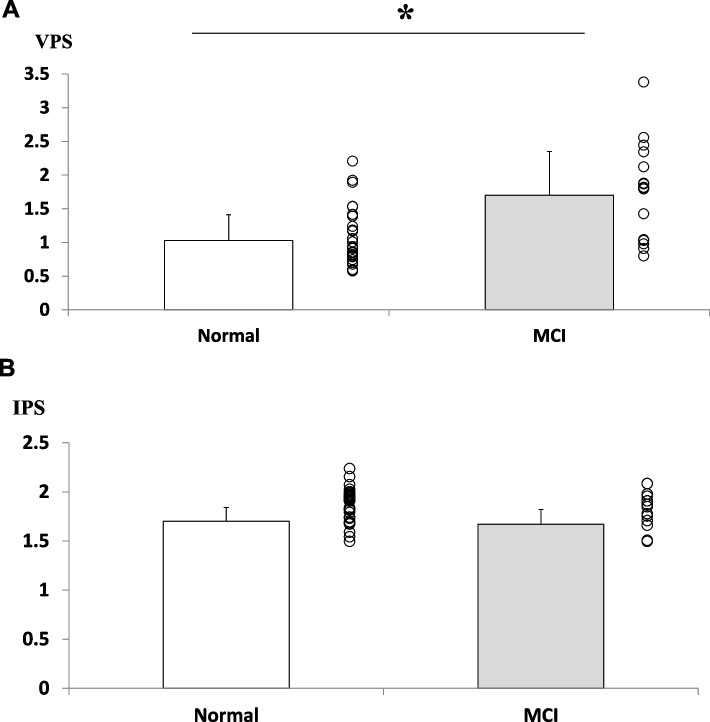
MCI group has significantly higher VPS. Comparison of the normal group and the MCI group is shown. Data are analyzed by the unpaired t-test. *p < 0.01. Bars, standard errors. MCI, mild cognitive impairment; IPS, index of postural stability; VPS, visual dependency index of postural stability

In accordance with the above result, when participants were categorized into two groups of normal (MoCA ≥ 26) and MCI (MoCA ≤ 25), MCI group showed significantly higher VPS values (Fig. 3A; mean difference: -0.67, 95% confidence interval: -1.04 to -0.29 by t-test), despite no differences in IPS values between the groups (Fig. 3B; mean difference: 0.03, 95% confidence interval: -0.06 to 0.12 by t-test). All the other items were not significantly different between the two groups (Table 1).

### ROC curve shows good sensitivity and specificity

**Fig. 4:**
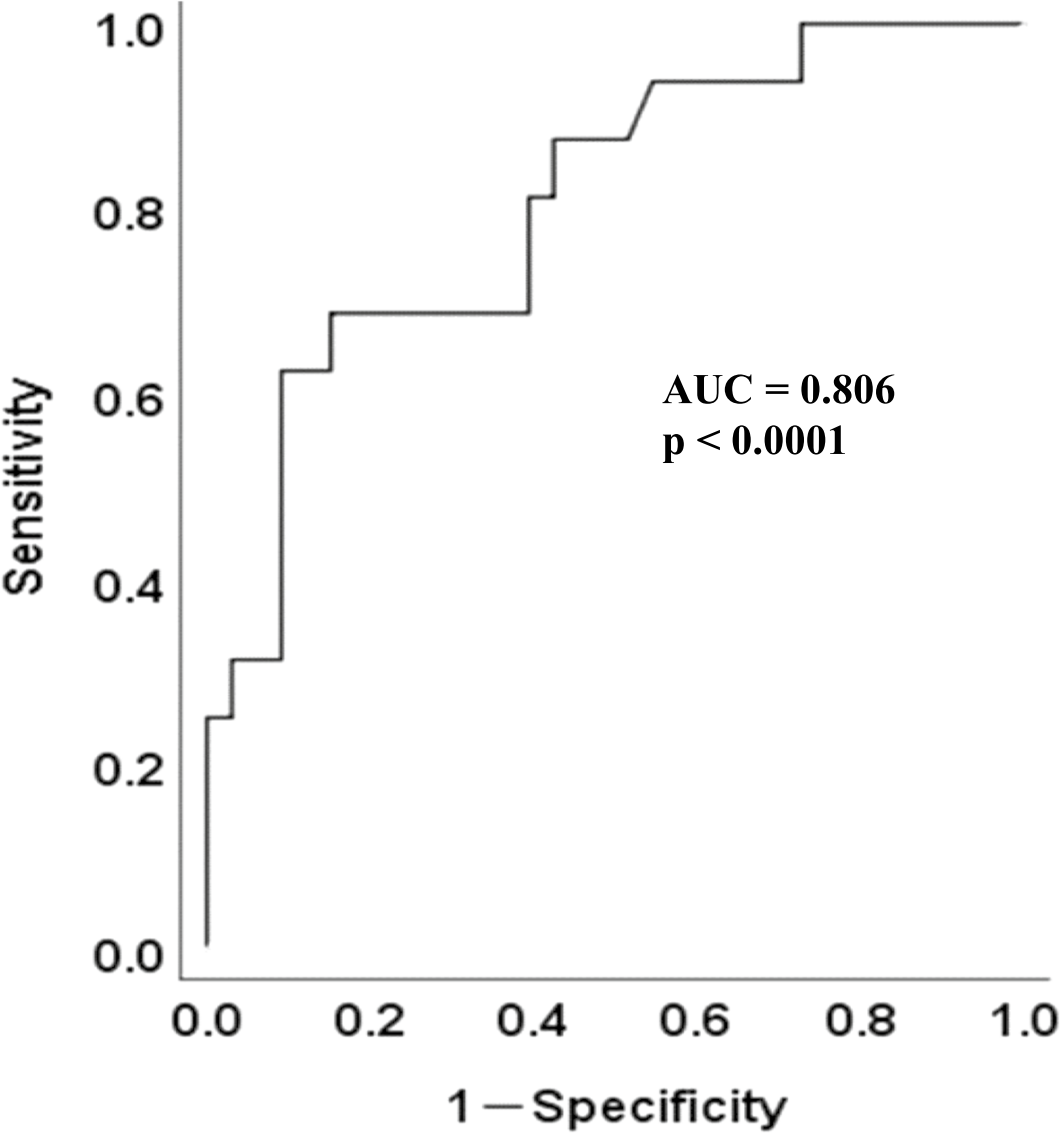
ROC curve shows good sensitivity and specificity. ROC curve of VPS is drawn to discriminate MCI from normal. ROC, receiver operating characteristic; VPS, visual dependency index of postural stability; MCI, mild cognitive impairment; AUC, area under the curve

The result of the receiver operating characteristic (ROC) analysis was shown in Fig. 4. As shown there, area under of the curve (AUC) was more than 0.8, indicating a good sensitivity and specificity in general. The VPS cutoff value obtained from the maximum value of the Youden Index was 1.00 (sensitivity; 81.3%, specificity: 57.6%, 95% confidential interval: 0.675 – 0.936). In other words, if it exceeds the cutoff value of 1.00, it is highly likely that it is MCI, and conversely, if it is below it, it is highly likely that it is not MCI.

## Discussion

In the present study, we suggested that our new balance indicator VPS is useful to screen individuals with MCI with high sensitivity.

The new indicator VPS (visual dependency index of postural stability) was created from the combination of IPS (index of postural stability) quantification method [17, 18] and foam posturography technique [13], both established previously. Among three sensory inputs critical for balance, namely vestibular, visual, and somatosensory inputs [16], the usage of foam rubber dampens the somatosensory input, and eye closure additionally eliminates visual input. Therefore, it is assumed that vestibular function can easily be assessed on foam rubber with eye-closed condition [13], and standing test with eyes closed on foam rubber has been well established as tests for clinical diagnosis of vestibular loss [34]. In fact, although we have not checked the point by ourselves, Fujimoto et al. examined the relationship between foam posturography data and direct vestibular function tests using cervical vestibular evoked myogenic potentials (cVEMPs), a well-established clinical test to examine vestibular function, and concluded that foam posturography on an eye-closed condition was useful for assessing vestibular impairment with abnormal cVEMPs [32]. Further taking the ratio between eye-closed and -opened conditions, we managed to raise the accuracy of measurement and named the value VPS. Since VPS is measured under the condition of getting almost no somatosensory and visual inputs as described above, its value is basically considered to reflect vestibular function.

Regarding the relationship between vestibular dysfunction and cognitive impairment, emerging evidence suggests that vestibular loss is disproportionately prevalent among individuals with MCI and dementia due to AD compared to healthy people [14, 15, 35]; in a study named Baltimore Longitudinal Study of Aging (BLSA), testing 183 healthy community-dwelling participants with a mean age of 72, they found that poorer vestibular function was significantly associated with poorer cognitive function assessed by several testing including TMT-B [14]. In addition, it has previously been shown that vestibular loss causes hippocampal atrophy and impaired spatial memory in humans [36]. A further study in BLSA found that poorer vestibular function was associated with significantly reduced hippocampal volume [37]. Thus, hippocampal atrophy may underlie the link between vestibular loss and cognitive decline.

As mentioned above, our method is based on the link between vestibular loss and cognitive decline. We think that this is the point of differentiation that creates extra value of our test in comparison to existing cognitive tests, because direct assessments of cognitive function can often present a psychological hurdle for subjects, which they would prefer to avoid if possible. In contrast, our method is not a direct assessment of cognitive function but just a balance ability test, so the psychological hurdle for subjects is much lower than direct methods.

Related to the link between vestibular and cognitive functions, hearing impairment is also reported to be linked with cognitive decline; in a small US prospective cohort study of 194 adults without baseline cognitive impairment and at least two brain MRIs with a mean of 19 years follow-up, it was reported that midlife hearing impairment measured by audiometry is associated with steeper temporal lobe volume loss, including in the hippocampus and entorhinal cortex [38]. The positive association between the loss of inner ear function and cognitive impairment is noteworthy.

Considering further in this regard, inner ear dysfunction and hippocampal atrophy might have some underlying pathological processes shared in common. The hippocampus has been known as one of the limited areas in the adult mammalian brain where neurogenesis normally occurs [39], and it is widely acknowledged that hippocampal neurogenesis is impaired in AD, which plays a role in cognitive decline [40, 41]. On the other hand, recent evidence suggests that reactive adult neurogenesis occurs in sensory systems following damages to the sensory nerve, and in fact this mechanism promotes balance recovery after vestibular loss [42]. Thus, it is conceivable that some common disorder related to neurogenesis in AD might underlie the link between inner ear dysfunction and hippocampal atrophy. Although currently this is just a hypothesis and further investigation is needed to prove it, this hypothesis might be related to the fact that AD is also strongly associated with olfactory dysfunction [43–46], given that olfactory bulb is another area of the adult brain where neurogenesis occurs vigorously [39] and people with AD exhibit smaller olfactory bulb volumes [47].

MCI is not just a prodrome to dementia, but a very important intervention point to treat AD. Recently, aducanumab, an antibody drug targeting Aβ [48], was approved by FDA in the US [49]. This decision made aducanumab the first new drug to be approved for the treatment of AD since 2003 and the first drug to ever be approved for modification of the course of AD. Since the drug is targeting MCI and early AD, the demand for efficient screening of MCI will become larger in the near future.

Because we started this project to realize an inexpensive and easy-to-use MCI screening system, we focused on WBB, a very popular Nintendo game machine, and successfully showed that it can be used as a good stabilometer that has the same performance as an authentic apparatus approved for the medical use. Nintendo has so far sold out more than 37 million WBB worldwide (https://www.nintendo.co.jp/ir/pdf/2009/091030.pdf). It means a huge potential for the novel approach to diagnose MCI at an earlier stage.

## Conclusions

We developed a new balance capability index termed VPS using WBB as a stabilometer. Early evidence suggests that this method is useful to screen individuals with MCI at an early and preclinical stage with high sensitivity.

## Supplementary Information

**Additional file 1: Supplementary Figure S1.** Correlations of age with eye-opened IPS on foam rubber. Scatterplot showing dynamic balance values in healthy individuals (*n* = 256). Lines depict mean, 68th, and 95th percentiles. Based on these data, age-predicted eye-opened IPS is calculated by -0.0003 × age^2^ + 0.0145 × age + 1.1602. **Supplementary Figure S2.** Correlations of age with eye-closed IPS on foam rubber. Scatterplot showing dynamic balance values in healthy individuals (*n* = 256). Lines depict mean, 68th, and 95th percentiles. Based on these data, age-predicted eye-closed IPS is calculated by -0.00006 × age^2^ - 0.0037 × age + 0.8805.

## Data Availability

All data generated or analyzed during this study are included in this published article and its supplementary information files.

## Abbreviations

AD: Alzheimer's disease
MCI: Mild cognitive impairment
VPS: Visual dependency index of postural stability
IPS: Index of postural stability
WBB: Wii balance board
MoCA: Montreal Cognitive Assessment
MMSE: Mini-Mental State Examination
TMT-A/-B: Trail Making Test parts A and B
CDR: Clinical dementia rating
