# Supplementary material for: New balance capability index as a screening tool for mild cognitive impairment
Source: BMC Geriatr. 2023 Feb 4;23:74. doi: 10.1186/s12877-023-03777-6 (PMC9899403; doi:10.1186/s12877-023-03777-6)
Supplement: Supplementary file 1 — Additional file 1: Supplementary Figure S1. Correlations of age with eye-opened IPS on foam rubber. Scatterplot showing dynamic balance values in healthy individuals (n = 256). Lines depict mean, 68th, and 95th percentiles. Based on these data, age-predicted eye-opened IPS is calculated by -0.0003 × age2 + 0.0145 × age + 1.1602. Supplementary Figure S2. Correlations of age with eye-closed IPS on foam rubber. Scatterplot showing dynamic balance values in healthy individuals (n = 256). Lines depict mean, 68th, and 95th percentiles. Based on these data, age-predicted eye-closed IPS is calculated by -0.00006 × age2 - 0.0037 × age + 0.8805. [file 12877_2023_3777_MOESM1_ESM.pdf]

Supplementary Information

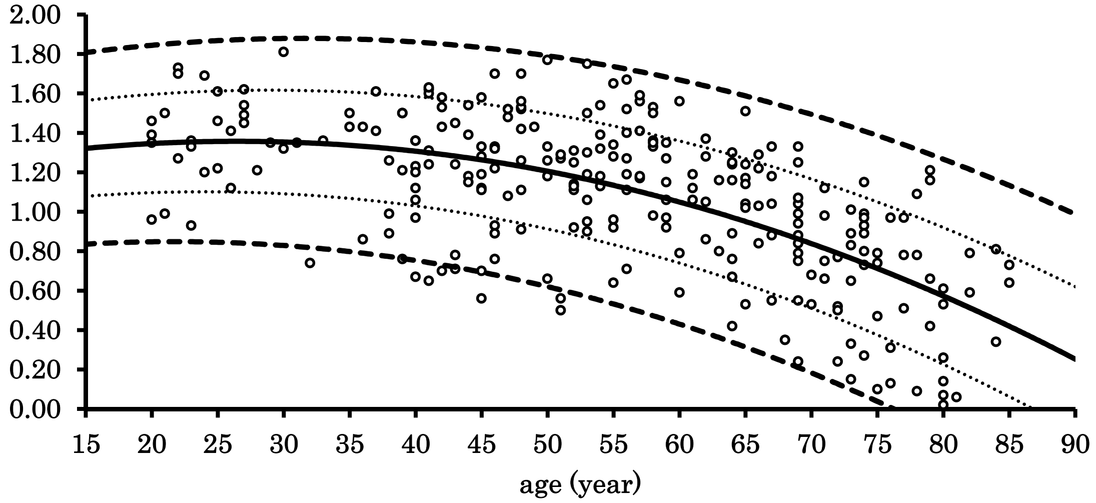

Supplementary Figure S1. Correlations of age with eye-opened IPS on foam rubber. Scatterplot showing dynamic balance values in healthy individuals (n = 256). Lines depict mean, 68th, and 95th percentiles. Based on these data, age-predicted eye-opened IPS is calculated by -0.0003 x age^2 + 0.0145 x age + 1.1602.

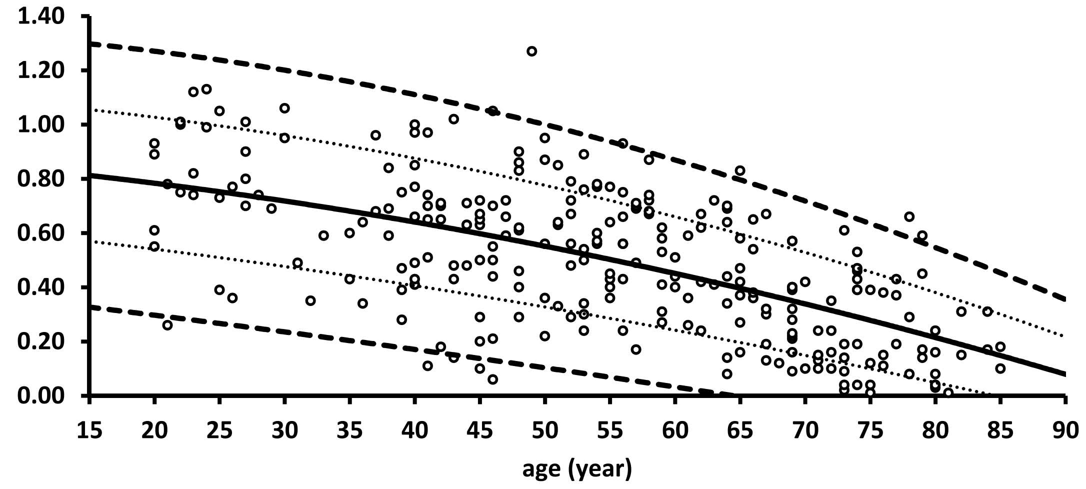

Supplementary Figure S2. Correlations of age with eye-closed IPS on foam rubber. Scatterplot showing dynamic balance values in healthy individuals (n = 256). Lines depict mean, 68th, and 95th percentiles. Based on these data, age-predicted eye-closed IPS is calculated by -0.00006 x age^2 - 0.0037 x age + 0.8805.
